# Orthographic and phonological contributions to flanker effects

**DOI:** 10.3758/s13414-020-02023-0

**Published:** 2020-06-03

**Authors:** Christophe Cauchi, Bernard Lété, Jonathan Grainger

**Affiliations:** 1 Laboratoire d’Étude des Mécanismes Cognitifs (EA 3082), Lyon 2 University, Lyon, France; 2 Laboratoire de Psychologie Cognitive, CNRS & Aix-Marseille University, 3 place Victor Hugo, 13331 Marseille, France; 3 Institute for Language Communication and the Brain, Aix-Marseille University, Marseille, France

**Keywords:** Reading, Flankers task, Phonology, Pseudohomophones

## Abstract

Does phonology contribute to effects of orthographically related flankers in the flankers task? In order to answer this question, we implemented the flanker equivalent of a pseudohomophone priming manipulation that has been widely used to demonstrate automatic phonological processing during visual word recognition. In Experiment 1, central target words were flanked on each side by either a pseudohomophone of the target (e.g., roze rose roze), an orthographic control pseudoword (rone rose rone), or an unrelated pseudoword (mirt rose mirt). Both the pseudohomophone and the orthographic control conditions produced faster and more accurate responses to central targets, but performance in these two conditions did not differ significantly. Experiment 2 tested the same stimuli in a masked priming paradigm and replicated the standard finding in French that pseudohomophone primes produce significantly faster responses to target words than orthographic control primes. Therefore, contrary to its impact on masked priming, phonology does not contribute to effects of flanker relatedness, which would appear to be driven primarily by orthographic overlap.

**Electronic supplementary material:**

The online version of this article (10.3758/s13414-020-02023-0) contains supplementary material, which is available to authorized users.

In a seminal study, Dare and Shillcock (2013) asked their participants to make lexical decisions to centrally located target words and nonwords while adding a subtle yet powerful twist to this classic paradigm. They added two letters to the left and two letters to the right of target stimuli, separated from the targets by a single space. These flanker letters could either be part of the target word (e.g., RO ROCK CK) or unrelated letters (e.g., PA ROCK TH). Participants could ignore the flanking letters because they were irrelevant for the task (nonword targets were also flanked by related and unrelated letters). Dare and Shillcock found that lexical decisions were facilitated by related flanker bigrams, not only when they respected their order in the target (as in the above example), but also when the order was reversed (e.g., CK ROCK RO). Crucially, they found the same amount of facilitation in these two conditions, a result that was to have important consequences for future theorizing.

In the present study, we examine the potential contribution of phonology to the effects of flanker relatedness obtained in the flankers task.^1^ Effects of flanker relatedness reported by Dare and Shillcock (2013), Grainger, Mathôt, and Vitu (2014), and Snell, Bertrand, and Grainger (2018) have been interpreted as reflecting the spatial integration of orthographic information spanning target and flankers. But in written languages like English and French, orthography is systematically confounded with phonology such that effects interpreted as being driven by orthographic overlap across target and flankers could in fact be driven by phonological overlap. That is, rather than the letters *R* and *O* in the word *ROCK*, it could be the phonemes /R/ and /o/ in /Rok/ that could be driving flanker effects. Indeed, the reduced effect of flanker relatedness found when switching letter order in the bigram flankers (e.g., OR ROCK KC; Grainger et al., 2014) could be interpreted as reflecting a disruption in phonological processing rather than a disruption in orthographic processing using ordered sequences of letters. That is, reversing letter order would hamper any attempt to generate a correctly ordered sequence of graphemes prior to their conversion into a string of phonemes. At the same time, the absence of an influence of bigram order (Dare & Shillcock, 2013; Grainger et al., 2014) is evidence against a strictly sequential encoding of graphemes and phonemes that is inherent in all accounts of phonological recoding. This therefore points to orthographic representations as the locus of effects of flanker relatedness, as hypothesized by Grainger et al. (2014). Indeed, Grainger et al. (2014) explained their findings and those of Dare and Shillcock (2013) in terms of flanker and target stimuli activating a common pool of sublexical orthographic representations (a bag-of-bigrams).^2^ According to Grainger and Ziegler (2011), the very nature of these bigram representations (ordered contiguous and noncontiguous letter combinations—referred to as “open-bigrams”) implies that they are dedicated to processing orthographic information and are not involved in encoding phonological information because the encoding of phonological information from letter strings requires more precise letter position information (see also Grainger, Dufau, & Ziegler, 2016). Therefore, according to this specific interpretation of the flanker results of Dare and Shillcock (2013), there should be no influence of flanker–target phonological overlap in the lexical decision version of the flankers task (flanking letters lexical decision). Moreover, given the evidence in favor of a key role for phonology in silent reading for meaning (e.g., Frost, 1998), and particularly in a language like French with relatively consistent spelling-to-sound mappings, although our prediction is a null effect, it is a strong prediction.

In order to provide a stricter test of the orthographic hypothesis, in the present study we measured effects of orthographic flanker relatedness while manipulating the amount of phonological overlap between flankers and targets (either flankers and targets shared all their phonemes in the correct order or they differed by at least one phoneme). We apply one particular manipulation that has played a central role in uncovering fast automatic phonological processes in visual word recognition—that is, creating pseudoword stimuli that would typically be pronounced identically to an existing word—so-called pseudohomophones. Using the masked priming technique (Forster & Davis, 1984), Ferrand and Grainger (1992) demonstrated that pseudohomophone primes facilitated lexical decisions to target words compared with nonhomophonic primes matched in orthographic overlap with targets (e.g., roze–ROSE vs. rone–ROSE). This result replicated the findings of Perfetti and Bell (1991) obtained in a paradigm that combines masked priming with a perceptual identification task, and both studies converged to show that prime duration must be sufficiently long (≈ 60 ms) in order to observe phonological priming over and above orthographic priming (see Ferrand & Grainger, 1993, for a time-course analysis; Grainger, Diependaele, Spinelli, Ferrand, & Farioli, 2003, for a replication; and Rastle & Brysbaert, 2006, for a review and meta-analysis). In Experiment 1, we test the same conditions as tested in Ferrand and Grainger (1992, 1993) and Grainger et al. (2003) in a flankers task, with prime stimuli becoming flanker stimuli that are repeated in the left and right positions.

## Experiment 1

### Method

#### Participants

Fifty-one students (45 females) from Lyon University, ranging in age between 18 and 29 years (mean age = 22 years, 1 month), gave informed consent to participate in this study. Participants were tested individually in a quiet room and reported being nondyslexic, native to the French language, and having normal or corrected-to-normal vision.

#### Design and stimuli

There were three flanker conditions (see Table 1). In the phonological condition (O+P+) the target was surrounded by pseudohomophone flankers (e.g., “roze rose roze”). In the orthographic-control condition (O+P−), the target was surrounded by the orthographic-control flankers that were pseudowords matched to the phonological flanker condition in terms of orthographic overlap with the target (e.g., “rone rose rone”). In the unrelated condition (O−P−), the target was flanked by pseudowords that shared no letters or phonemes with the target (e.g., “fuli rose fuli”). A Latin-square design was used such that each target appeared in only one condition for one participant, but in all conditions across all participants.

**Table 1:** Example of word and pseudoword targets (center) and flanker stimuli in the three experimental conditions: pseudohomophone flankers (O+P+), orthographic-control flankers (O+P−), unrelated flankers (O−P−)

Conditions	Word	Pseudoword
O+P+	roze rose roze	voze vose voze
O+P−	rone rose rone	vone vose vone
O−P−	fuli rose fuli	huna vose huna

A set of 108 French words and a set of 108 pseudowords served as target items in this Experiment. These 216 target items were four to five letters long (average length = 4.6 letters). Words were selected from the MANULEX lexical database (Lété, Sprenger-Charolles, & Colé, 2004). The mean frequency of these words in Zipf values (van Heuven, Mandera, Keuleers, & Brysbaert, 2014) was 5.1 (range: 3.5–6.5), qualified as high lexical frequency according to MANULEX. The main criterion for word selection was that both a pseudohomophone and a matched orthographic control pseudoword could be created, both differing by a single letter at the same position from the corresponding target word. Finally, a set of 108 unrelated pseudoword flanker stimuli were constructed that differed maximally from the corresponding target word both orthographically and phonologically, while respecting the orthotactic and phonotactic constraints of French. The pseudoword targets were created from the set of word targets by a single letter substitution, and the flankers were created using the same procedure as for the word targets. There were 36 target words and 36 target pseudowords in each condition. An overview of the experimental conditions is provided in Table 1 (see the Appendix Table 4 for a full list of the materials).

#### Procedure

**Fig. 1:**
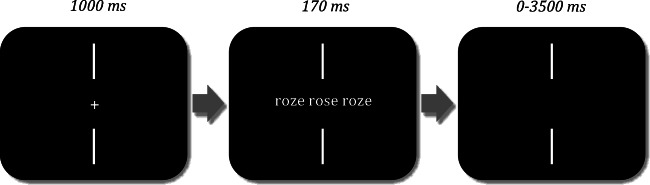
Description of the procedure of Experiment 1. In this example, the target word ‘rose’ is flanked by the pseudohomophone flankers ‘roze’. After the fixation cross, target and flankers appear onscreen for 170 ms, and centred with respect to the vertical fixation bars. After the stimuli disappear, participants have a maximum of 3,500 ms to indicate whether the central stimulus was a word or not

The experiment was implemented with OpenSesame (Mathôt, Schreij, & Theeuwes, 2012). Stimuli were presented on an HP ProBook 640 G2 monitor calibrated in 18-inch (1,366 × 768 px, 80 Hz). Stimuli were displayed in lowercase Courier New font (19 pts) in white on a black background. At a viewing distance of 40 cm, each character subtended approximately 0.33 degrees of visual angle. Manual responses were collected with the computer keyboard. Each trial started with two vertical fixation bars above and below a centralized fixation cross. After 1,000 ms, the central fixation cross disappeared, and the target (a word or a pseudoword), flanked by two pseudowords on each side, was presented between the two vertical fixation bars (see Fig. 1). After 170 ms, the stimulus was blanked. Participants indicated as quickly and accurately as possible whether the target was a word or a pseudoword by pressing the right or the left button (‘q’ and ‘m’, respectively, on an AZERTY keyboard). The experiment lasted approximately 15 minutes. The 216 trials were divided into four blocks of 54 trials. The different blocks and the trials within a block were presented in a different random order for each participant. The task began with 36 practice trials followed by the main experiment.

### Results

Only the data for word targets were analyzed. We first retained all trials with a response time (RT) lying between 300 and 3,000 milliseconds. The analyses of RTs excluded incorrectly answered trials for all stimuli (5.03%). Additionally, both the RTs and the error-rate analyses excluded trials with an RT beyond 2.5 standard deviations from the mean for each participant (2.49%). Data were analyzed in the R statistical computing environment (Core Team, 2018) using linear-mixed-effects models (LMEs), with items and participants as crossed random effects, fitted with the lmer function from the lme4 package (Version 1.1-21; Bates, Maechler, Bolker, & Walker, 2015). The maximal random effects structure that converged was one including by-participant and by-item random intercepts. We conserved the same structure both for the RT and error rate analyses. The *b* values, standard errors (*SE*s) and *t* values (RTs) or *z* values (errors) are reported, with *t* and *z* values beyond |1.96| deemed significant (Baayen, 2008). A complementary Bayes factor analysis was performed using the lmBF function from the BayesFactor package (Version 0.9.12-4.2; Morey & Rouder, 2018). Each model was compared with the intercept-only model. Along with the LME analyses, we report the results of the Bayes factor analysis and the interpretation recommended by Lee and Wagenmakers (2013). For all significant effects in the LME analyses, we report the value of BF_10_ (evidence for H_1_ against H_0_), and we report BF_01_ (evidence for H_0_ against H_1_) for all nonsignificant effects.

Mean RTs per condition are presented in Table 2. The following analyses were conducted taking the unrelated condition as a reference. The phonological condition O+P+ was significantly faster than the unrelated condition O−P− in the RT analysis (*b* = −20.72, *SE* = 3.42, *t* = −6.06; BF_10_ > 100, extreme evidence for H_1_), and the error rate was significantly decreased in the phonological condition as compared with the unrelated condition (*b* = −0.73, *SE* = 0.16, *z* = −4.48; BF_10_ > 100, extreme evidence for H_1_). The orthographic-control condition O+P− was significantly faster than the unrelated condition in the RT analysis (*b* = −26.53, *SE* = 3.43, *t* = −7.74; BF_10_ > 100, extreme evidence for H_1_), and the errors rate was significantly lower in the orthographic control condition (*b* = −0.59, *SE* = 0.16, *z* = −3.7; BF_10_ = 41, very strong evidence for H_1_). In planned comparisons, we also analyzed the critical contrast between the phonological and orthographic-control conditions and found no significant difference between these two conditions in both the RT analysis (*b* = 5.95, *SE* = 3.37, *t* = 1.77; BF_01_ = 7, moderate evidence for H_0_) and the error rate analysis (*b* = −0.16, *SE* = 0.18, *z* = −0.88; BF_01_ = 22, strong evidence for H_0_). Although numerically fewer errors were made in the phonological flanker condition, the RTs were actually longer in that condition.

**Table 2:** Mean RTs (in milliseconds) and errors rates (probabilities) for word targets (standard deviations in parentheses) accompanied by pseudohomophone flankers (O+P+), orthographic-control flankers (O+P−), or unrelated flankers (O−P−) in Experiment 1

Conditions	RT	Error rate
O+P+	620 (69)	.038 (.042)
O+P*−*	613 (64)	.042 (.033)
O*−*P*−*	640 (66)	.071 (.063)

### Discussion

The results of Experiment 1 are straightforward. We found highly robust effects of orthographically related flankers on both RTs and error rates, with both the phonological flanker and the orthographic control flankers generating faster and more accurate responses to central target words compared with the unrelated flanker condition. On the other hand, the phonological and orthographic control conditions did not differ significantly, implying that phonological flanker–target overlap was not contributing to the effects of orthographically related flankers.

However, before concluding that phonology does not contribute to the effects seen with orthographically related flankers, we need to demonstrate that the same stimuli exhibit the standard pattern of phonological priming effects reported in prior studies. To do so, we tested exactly the same stimuli in a masked priming experiment where flanker stimuli became prime stimuli accompanying the same set of target words and pseudowords.

## Experiment 2

### Method

#### Participants

Forty-five students (42 females) from Lyon university, ranging in age between 18 and 23 years (mean age = 20 years 7 month), gave informed consent to participate in this study. In this experiment, the participants were tested individually in a quiet room and reported being nondyslexic, native to the French language, with normal or corrected-to-normal vision.

#### Design and stimuli

The design and stimuli were the same as in Experiment 1, except that flanker stimuli now become prime stimuli in a masked priming experiment.

#### Procedure

**Fig. 2:**
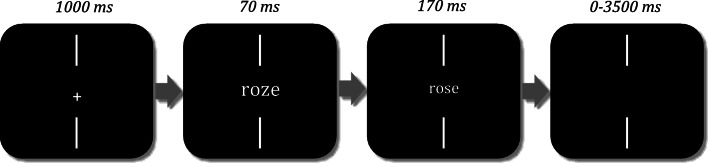
Priming procedure used in Experiment 2. In this example, the target word ‘rose’ is primed by the pseudohomophone ‘roze’ in the phonological (O+P+) priming condition. The prime appears onscreen during 70 ms between two vertical bars directly followed by the target display for 170 ms. Note the change in size between prime and target in order to minimize purely visual overlap with related primes. After the target disappeared, participants had a maximum of 3,500 ms to indicate whether it was a word or a nonword

Stimuli were displayed in lowercase Courier New font, in white on a black background. To avoid orthographic overlap between primes and targets, we applied different font size between these two. In this way the prime font size was 9 pts greater than the target font size, which was the same font size as the target and the flankers in Experiment 1 (19 pts). At a viewing distance of 40 cm, the prime and target characters subtended respectively 0.45 and 0.33 degrees of visual angle. Each trial started with two vertical fixation bars above and below a centralized fixation cross. After 1,000 ms, the prime was briefly presented for 70 ms^3^ between the two vertical fixation bars, directly followed by the target presentation (a word or a pseudoword) for 170 ms (see Fig. 2). Target duration was the same as the flanker–target duration used in Experiment 1. Then, the target was blanked. Participants indicated as quickly and accurately as possible whether the target was a word or a pseudoword, by pressing the right or the left button (‘q’ and ‘m’, respectively, on an AZERTY keyboard). There were three priming conditions that mimicked the three flanker conditions of Experiment 1, with the flanker stimuli becoming primes. The experiment lasted approximately 15 minutes.

### Results

We applied criteria identical to those used in Experimental 1 for the exclusion of trials in Experiment 2. The analyses of RTs excluded incorrectly answered trials for all stimuli (5.66%). Additionally, both the RTs and the error rate analyses excluded trials with an RT beyond 2.5 standard deviations of the grand mean for each participant (2.94%). Mean RTs per condition are presented in Table 3. We again employed LMEs for the analysis of RTs and error rates. We used models with the maximal random structure that successfully converged. For the analysis of RTs, this was a model that included by-participant random intercepts and slopes and by-item random intercepts. The analysis of errors was performed with a model that included by-participant random intercepts and by-item random intercepts and slopes. We report *b* values, standard errors (*SE*s), and *t* values (RTs) or *z* values (errors), with *t* and *z* values beyond |1.96| deemed significant. We also report Bayes factor analyses as in Experiment 1.

**Table 3:** Mean RTs (in milliseconds) and error rates (probabilities) for word targets (standard deviations in parentheses) preceded by pseudohomophone primes (O+P+), orthographic-control primes (O+P−), or unrelated primes (O−P−) in Experiment 2

Conditions	RT	Error rate
O+P+	622 (62)	.057 (.045)
O+P*−*	636 (63)	.056 (.052)
O*−*P*−*	643 (69)	.056 (.061)

The following analyses were conducted taking the unrelated condition as reference in the model. The phonological condition was faster than the unrelated condition in the RT analysis (*b* = −20.52, *SE* = 3.51, *t* = −5.83; BF_10_ > 100, extreme evidence for H_1_). Error rates did not differ between these two conditions (*b* = −0.04, *SE* = 0.22, *z* = −0.19; BF_01_ = 25, strong evidence for H_0_). However, contrary to Experiment 1, the difference between the orthographic-control condition and the unrelated condition was not significant in the RT analysis (*b* = −7.29, *SE* = 3.9, *t* = −1.87; BF_01_ = 6, moderate evidence for H_0_) as well as in the error-rate analysis (*b* = −0.09, *SE* = 0.24, *z* = −0.38; BF_01_ = 25, strong evidence for H_0_). By restricting the analyses to the phonological and orthographic-control conditions (conditions of interest), we found that RTs in the phonological condition were significantly faster compared with the orthographic-control condition (*b* = −13.42, *SE* = 3.51, *t* = −3.82; BF_10_ = 6, moderate evidence for H_1_). Error rates did not differ significantly between these two conditions (*b* = −0.08, *SE* = 0.26, *z* = 0.3; BF_01_ = 24, strong evidence for H_0_).

### Discussion

The results of Experiment 2 perfectly replicate the findings reported by Ferrand and Grainger (1992, 1993) and Grainger et al. (2003) with a larger sample of stimuli and a larger sample of participants. These finding therefore provide additional support to the meta-analysis of Rastle and Brysbaert (2006) and reinforce their conclusion that pseudohomophone priming effects are indeed ‘real’. Most important, however, is that the results of Experiment 2 confirm that the absence of an effect of phonological flanker overlap in Experiment 1 was not due to poor stimulus selection. However, given that the key result of Experiment 1 is a null effect, in order to provide a stronger test of the absence of an effect of phonological target–flanker overlap in that experiment, we performed a combined analysis of Experiments 1 and 2 in order to test for an interaction with task (flankers vs. priming).

## Combined analysis

In the combined analysis, the three flanker/priming conditions tested in Experiments 1 and 2 were included as a within-participant factor, and task (flankers vs. priming) was included as a between-participant factor.

### Results

**Fig. 3:**
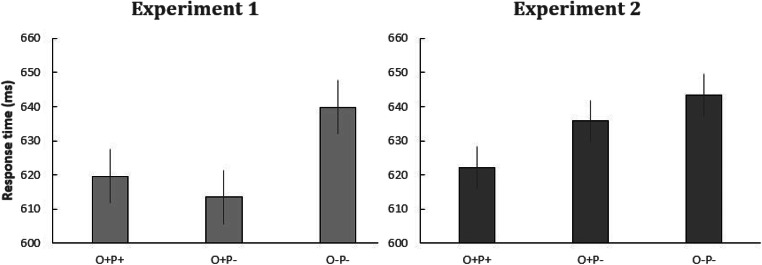
Mean RTs in the pseudohomophone (O+P+), orthographic control (O+P*−*), and unrelated (O*−*P*−*) conditions in Experiment 1 (flankers task) and Experiment 2 (priming). Error bars depict 95% confidence intervals

We again employed LMEs for the analyses of RTs and error rates using the maximal random structure that successfully converged. For the analysis of RTs, this was a model that included by-participant and by-item random intercepts. The analysis of errors was performed with a model that included by-participant random intercepts and by-item random intercepts and slopes. A Bayes factor analysis was also applied. In the phonological effects analysis, there was a significant interaction between condition (O+P+, O+P−) and task (flankers task, priming task), with a +14-ms phonological effect in the priming task compared with a −7-ms effect in the flankers task (*b* = −19.54, *SE* = 4.9, *t* = −3.99; BF_10_ = 7, moderate evidence for H_1_). The interaction was not significant in the error rates (*b* = −0.20, *SE* = 0.24, *z* = 0.83; BF_01_ = 31, very strong evidence for H_0_). In the orthographic effects analysis there was a significant interaction between condition (O+P−, O−P−) and task (flankers task, priming task), with a +7-ms orthographic effect in the priming task compared with a +27-ms effect in the flankers task (*b* = 19.35, *SE* = 4.9, *t* = 3.94; BF_10_ = 61, very strong evidence for H_1_). The interaction was also significant in the error rates (*b* = −0.55, *SE* = 0.23, *z* = 2.4), but the Bayes factor analysis yielded no evidence for this effect (BF_10_ = 0.99). The condition means for RTs are shown in Fig. 3.

### Discussion

The combined analysis of the results of Experiments 1 and 2 provide unequivocal evidence that effects driven by phonological information differ in the flankers task compared with masked priming. The significant interaction between the phonological contrast (O+P+, O+P−) and task indicates that effects of phonological overlap were significantly greater in priming. This provides further evidence that the absence of an effect of target–flanker overlap in Experiment 1 was not due to a lack of power or poor stimulus selection. Interestingly, the combined analysis also revealed a greater impact of orthographic overlap (O+P−, O−P−) in the flankers task relative to masked priming. This provides further support in favor of Grainger et al.’s (2014) interpretation of Dare and Shillcock’s (2013) flanker effects as reflecting the spatial integration of orthographic information.

## General discussion

Experiment 1 was designed to test for effects of flanker–target phonological overlap in the flankers task. Prior research had manipulated flanker–target orthographic overlap (e.g., Dare & Shillcock, 2013; Grainger et al., 2014; Snell, Bertrand, & Grainger, 2018) and concluded that the observed flanker effects were being driven by the spatial integration of orthographic information across flanker and target stimuli (Grainger et al., 2014; see Grainger, 2018, for a review). However, in all these studies flanker–target orthographic overlap was confounded with flanker–target phonological overlap, and therefore it might be that phonology was the source of effects of flanker relatedness. In this respect, it is important to note that Grainger et al. (2014) proposed that the spatial integration of orthographic information is achieved via open-bigram representations (Grainger & van Heuven, 2004; Whitney, 2001). Now, in the general framework for orthographic processing proposed by Grainger and Ziegler (2011); see also Grainger et al., 2016), open-bigram representations are dedicated to mapping letter-level representations onto whole-word orthographic representations and are not assumed to provide an appropriate code for associating letters with sounds (i.e., phonological processing). Therefore, we predicted that phonological information is not integrated across stimuli in the flankers task. The results of Experiment 1 are in line with this prediction.

Experiment 2 was performed simply in order to check that the same stimuli tested in the flankers task of Experiment 1 would indeed show phonological effects in masked priming. This was found to be the case. Crucially, a combined analysis of the two experiments revealed a significant interaction between task (flankers vs. priming) and the contrast between pseudohomophones and orthographic controls. Phonological priming effects were significantly greater in masked priming than in the flankers task. Interestingly, the combined analysis also revealed that the contrast between orthographic controls and the unrelated condition was significantly greater in the flankers task than in masked priming (see Fig. 3). Both of these patterns clearly point to a key role for orthographic representations in the spatial integration of information extracted in parallel from target and flanker stimuli in the flankers task. In the framework proposed by Grainger et al. (2014), these orthographic representations are open-bigrams (Grainger & van Heuven, 2004; Whitney, 2001), which are not suited for accurately generating a phonological representation from print, hence the absence of phonological effects in the flankers task in Experiment 1.

How might alternative theories of orthographic processing account for the present findings? Three of the major models in this field, Davis’ (2010) spatial coding model, the Bayesian reader (Norris, 2006), and the overlap model (Gomez, Ratcliff, & Perea, 2008), all assign a flexible position to each individual letter identity in a string of letters. The assigned flexibility is chosen to be just enough to enable such models to capture transposed-letter effects (see Grainger, 2008, for a review) without creating havoc in normal word recognition. Just how such letter-position coding schemes can be applied to simultaneously process multiple stimuli has yet to be determined, and as such, Dare and Shillcock’s (2013) findings remain a challenge for these models. Nevertheless, whatever the proposed mechanism, one important point remains: contrary to Grainger and Ziegler’s (2011) model, the exact same letter representations are involved in orthographic processing and phonological recoding in these alternative models. Therefore, any adaptation of these models to account for Dare and Shillcock’s (2013) findings will necessarily predict that flanker phonology effects should be obtained in conditions where orthographic effects are obtained.

Concerning the role of phonology in silent reading for meaning, it is important to point to the evidence that phonological representations can be activated by parafoveal stimuli, so it is not the eccentricity of the flanker stimuli that is cancelling the phonological effect in Experiment 1. Sentence reading experiments using the boundary technique (Rayner, 1975) have repeatedly shown that parafoveal previews that are phonologically related to the upcoming target word facilitate reading of that word compared with orthographic control previews. This is the case for both homophone previews (e.g., Bélanger, Mayberry, & Rayner, 2013; Chace, Rayner, & Well, 2005; Pollatsek, Lesch, Morris, & Rayner, 1992) and pseudohomophone previews (e.g., Miellet & Sparrow, 2004; see Vasilev, Yates, & Slattery, 2019, for a review and meta-analysis). On the other hand, Tiffin-Richards and Schroeder (2015) failed to observe a preview effect with pseudohomophones in their adult participants, with the effects only being significant in children. This raises the possibility that phonological flanker effects might be observable in beginning readers, given that it is well established that phonology plays a key role in the process of reading development (Grainger, Lété, Bertrand, Dufau, & Ziegler, 2012; Share, 1995; Ziegler, Perry, & Zorzi, 2014). Counter to this, however, is the finding reported by Chace et al. (2005) that only skilled readers show phonological parafoveal preview effects. Furthermore, although Bélanger et al. (2013) found effects of homophone previews in both skilled and less-skilled readers, they reported that the effects were only robust when the preview was a high-frequency word. On the other hand, there is evidence that phonological priming is not affected by word frequency (e.g., Lukatela & Turvey, 1994).^4^

Why is there evidence for phonological parafoveal preview effects and no evidence for phonological flanker effects? In preview experiments, the preview and target occupy the same spatiotopic location along a line of text, and this enables integration of orthographic, phonological, semantic, and syntactic information at a given location (Snell, Meeter, & Grainger, 2017). Therefore, phonological information is indeed processed in the parafovea, but is not integrated within the central channel for word identification, as postulated in the work of Grainger et al. (2014) and Snell, Meeter, and Grainger (2017). However, failure to integrate phonological information across adjacent stimuli does not imply that phonological information is not processed within the central processing channel. Phonology has to be processed within the central processing channel (e.g., Grainger et al., 2016) in order to account for phonological influences on single-word reading and sentence reading (Frost, 1998; Rastle & Brysbaert, 2006; Vasilev et al., 2019). The limitation of this processing relative to orthographic information is that it is tied to a specific spatiotopic location in a line of text. Our model therefore predicts that, contrary to phonological preview effects seen in sentence reading with the boundary technique, phonological parafoveal-on-foveal effects should not be observed in the same paradigm. That is, when reading the sentence ‘The boy picked the rose from the garden’, reading times of the target word ‘rose’ should not be influenced by the replacement of the parafoveal word ‘from’ with a pseudohomophone ‘roze’ prior to the eyes moving to that location. This predicted null effect could be contrasted with well-established orthographic parafoveal-on-foveal effects obtained during sentence reading (Angele, Tran, & Rayner, 2013; Dare & Shillcock, 2013; Mirault et al., 2020; Snell, Vitu, & Grainger, 2017). Such additional experimentation would further help connect the results obtained with the flankers task and those obtained in more natural reading situations (for a recent debate, see Snell & Grainger, 2019; and accompanying commentaries, Schotter & Payne, 2019; White, Boynton, & Yeatman, 2019).

Finally, one other issue concerns whether the absence of phonological flanker effects reported in the present study might be specific to French. The French language is characterized by very consistent spelling-to-sound mappings and inconsistent sound-to-spelling mappings. According to the orthographic depth hypothesis (Katz & Frost, 1992; see also Frost, 1998), it is the consistency in spelling-to-sound mappings that is critical in determining the relative involvement of phonological representations in the process of silent reading in a given language. Therefore, given that French is very consistent in this respect, we expect the null effect of flanker phonology to generalize to other alphabetic languages, and we suspect that the slightly greater consistency found in some languages such as Finnish and Italian should not make a significant difference. Of course, it might be possible that it is the inconsistent mapping of sound-to-spelling in French that causes the absence of flanker phonology effects. But then such an account of flanker effects would have to explain why the exact same stimuli produced robust phonological priming effects in the present study.

In sum, in the present work, using the flankers task, we have shown that while target–flanker orthographic overlap has a major impact on central target word processing, as attested by prior work, target–flanker phonological overlap has no influence whatsoever. This pattern contrasts with the findings obtained when the phonological relation concerns stimuli associated with the same spatiotopic location, as is the case in priming studies with central presentation of prime and target, and in parafoveal preview studies. Future research could confirm this null effect of integration of phonological codes across distinct spatiotopic locations using a parafoveal-on-foveal manipulation during sentence reading.

### Electronic supplementary material

ESM 1 (XLSX 905 kb)

## Appendix

**Table 4:** List of target words and flankers in each experimental condition

Target	Flanker conditions		
	O+P+	O+P−	O−P−
âgée	âger	âged	remp
aîné	aîner	aînej	carom
ainsi	einsi	oinsi	jeuve
après	aprai	aproi	veuco
assez	açez	atez	dilo
assis	açis	apis	lepo
aussi	ossi	essi	lenu
avant	avent	avint	corge
bébé	béber	bében	sajoi
belle	baile	boile	tidra
blanc	blans	blano	sutiz
brin	brein	bruin	osopa
café	cafer	cafel	piulo
cage	caje	cate	hirm
carte	karte	larte	funol
cassé	caçé	caté	pode
cause	cauze	caude	groid
chose	choze	chole	jabat
cinq	cink	cinf	ploj
clair	clère	clure	bouve
clef	cler	clir	pado
coin	koin	toin	galm
comme	komme	domme	biant
corde	korde	porde	blait
côte	caute	caote	saril
coup	koup	joup	dati
cour	kour	gour	maip
doigt	doix	doir	braf
douze	douse	doure	aimbi
eaux	haux	haox	sicy
écran	ékran	ésran	tumpe
enfin	anfin	onfin	datsu
entre	antre	intre	bloic
envie	anvie	onvie	aphys
étage	étaje	étaze	fusox
faim	fain	faig	sorl
faute	fôte	fite	mird
film	fylm	folm	erat
forêt	forai	forau	lapas
frein	frin	frun	dola
froid	froix	froil	acant
fusée	fuzée	fubée	apant
fusil	fuzil	funil	ogran
futé	futer	futek	kolia
genre	jenre	senre	clomb
gosse	goçe	gope	piun
grain	grein	gruin	ousto
grand	grend	grond	bieux
grise	grize	grite	afant
habit	habie	habif	englo
hibou	hybou	hebou	raseu
idée	ider	iden	frou
jaune	jône	june	gofu
joli	joly	jolu	buat
lampe	lempe	lompe	umori
large	larje	larne	dunil
liste	lyste	luste	fopra
main	mein	muin	eslu
mais	maie	maig	coum
mange	manje	manre	doils
mémé	mémer	mémur	olsar
mois	moie	moik	kenu
moto	motau	motai	phida
musée	muzée	munée	polsa
noix	nois	noiq	trof
nord	nore	nory	filp
nuage	nuaje	nuare	ploid
objet	objai	objui	asupi
oncle	onkle	onsle	tidas
orage	oraje	orafe	imnuz
page	paje	pame	coir
pain	pein	puin	solc
parmi	parmy	parmo	doste
part	pare	parl	lebi
passe	pace	pave	clod
peau	paux	pauf	fril
pépé	péper	pépen	sanic
piste	pyste	puste	cerlo
plage	plaje	plare	riroc
plan	plen	plun	sart
plein	plin	plef	guca
pose	poze	poje	fima
près	prai	pran	libe
quand	quend	quind	lompe
radio	radyo	radeo	centu
reine	raine	roune	hacot
rôle	raule	riule	kimpa
rose	roze	rone	fuli
rosée	roser	rosen	guici
rouge	rouje	roude	labis
seau	ceau	deau	kiln
selon	celon	felon	ragui
série	cérie	dérie	alvom
sirop	sirau	siran	camde
stylo	stilo	stulo	jaive
sucre	sukre	sudre	ploin
sujet	sujai	sujat	ainco
tapis	tapys	tapus	enbul
télé	téler	téleu	acuid
tissu	tiçu	tipu	olan
train	trein	truin	glove
très	trai	tran	glad
vase	vaze	vade	quzi
vent	vant	vunt	rial
voici	voicy	voicu	anent
volé	vaulé	vanlé	harun
vous	voux	voul	diny
vrai	vret	vrun	omse

## Footnotes

1 In prior work, we have used the terms “flanker paradigm” or “flanker task”. Here, we adopt the original terminology of Eriksen (e.g., Eriksen, 1995).

2 It is the concept of an unordered set (a “bag”) of ordered letter combinations that allows this particular model to account for Dare and Shillcock’s (2013) findings.

3 The choice of prime duration was determined by prior research in French using similar materials (Ferrand & Grainger, 1992, 1993; Grainger et al., 2003) showing that pseudohomophone priming effects require prime durations in the range of 60–70 ms in order to emerge.

4 In post hoc analyses, we examined the impact of word frequency on both flanker and priming effects by adding Zipf frequency as a continuous variable in the LME analyses. Although word frequency did significantly affect RTs and errors in both Experiments 1 and 2, it did not interact with either flanker or priming effects (all *t*s < 1.4; all *z*s < 1.2).
